# Anti-cancer activity of an ethanolic extract of red okra pods (*Abelmoschus esculentus* L. Moench) in rats induced by *N*-methyl*-N*-nitrosourea

**DOI:** 10.14202/vetworld.2022.1177-1184

**Published:** 2022-05-12

**Authors:** Manikya Pramudya, Firli Rahmah Primula Dewi, Richard W. Wong, Devinta Wahyu Anggraini, Dwi Winarni, Sri Puji Astuti Wahyuningsih

**Affiliations:** 1 Department of Biology, Faculty of Science and Technology, Universitas Airlangga, Campus C, Mulyorejo, Surabaya, Indonesia; 2 Cell Bionomics Research Unit, Institute for Frontier Science Initiative, Kanazawa University, Kanazawa 920-1192, Japan

**Keywords:** cancer, cytokines, histology of mammary gland, *N*-methyl-*N*-nitrosourea, red okra pods

## Abstract

**Background and Aim:**

Breast cancer is the most frequent malignancy in women. The consumption of phytochemical components from plants may play an essential role in preventing and treating this cancer. This study aimed to investigate the anti-cancer activity of an ethanolic extract of red okra pods (EEROP) in rats (*Rattus norvegicus*) induced by *N*-methyl- *N*-nitrosourea (MNU).

**Materials and Methods:**

The experimental animals were divided into six groups (n=5/group), namely, KN (normal control, without any treatment), K− (negative control, exposed to MNU without EEROP), K+ (positive control, exposed to MNU and Methotrexate), and the treatment Groups P1, P2, and P3 (exposed to MNU and EEROP at doses of 50, 100, and 200 mg/kg body weight [BW], respectively). Intraperitoneal delivery of MNU and EEROP oral administration was carried out for 8 weeks. After the end of treatment, the parameters of cytokines, CD4+ and CD8+ T cells, and mammary gland histology were measured.

**Results:**

The results showed that EEROP at doses of 100 and 200 mg/kg BW significantly downregulated interleukin (IL)-6, IL-1ß, tumor necrosis factor (TNF)-α, IL-17, IL-10, and tumor growth factor-β (p<0.05). In addition, doses of 200 mg/kg BW significantly increased the activity of CD4+ and CD8+ T cells, prevented the proliferation of mammary gland epithelial cells, and yielded a significantly thinner epithelium of the mammary gland (p<0.05).

**Conclusion:**

It can be concluded that EEROP was an effective anti-cancer agent by modulating the immune response. Further studies using a nanoparticle system are warranted to achieve optimal working conditions for these bioactive compounds.

## Introduction

Breast cancer is the most frequent malignancy in women. In 2018, researchers estimated that approximately one new case of breast cancer was diagnosed every 18 s and that 626,679 women died from breast cancer [1]. The global incidence of breast cancer increased by 3.1% annually, from 641,000 cases in 1980 to over 1.6 million cases in 2010. During the last 10-15 years, the concept of breast cancer treatment evolved by emphasizing biological therapies, to reduce the side effects of conventional treatments [2]. The mechanisms of initiation of breast cancer are unknown; however, much effort has been made to characterize breast cancer molecularly and describe its formation and development. Inflammation by environmental exposure to substances, such as carcinogenic agents, can promote cancer development and progression [3], and the inflammatory microenvironment is an essential component of tumor formation [4]. Therefore, the inflammatory immune response is widely detected in individuals with cancer. In breast cancer, the presence of tumor-infiltrating lymphocytes with a high CD4+/CD8+ ratio is an indicator of poor prognosis [5]. The expression of cytokines and chemokines in the tumor microenvironment can either promote or inhibit tumor development and progression [6]. Interferon-gamma (IFN-γ) and IL-10 are cytokines that can control the immune and inflammatory environment, thus acting as anti-tumor molecules [3,6]. Conversely, IL-1, IL-6, tumor necrosis factor (TNF)-α, and tumor growth factor-β (TGF-β) can trigger tumor progression [3,7].

The consumption of phytochemical components from plants may play an essential role in preventing and treating many diseases [8]. Okra (*Abelmoschus esculentus* L. Moench) is a vegetable plant belonging to the *Malvaceae* family that is widely consumed in various countries [9]. Because of its extensive medicinal value, okra has been used to treat many ailments [10]. The *in vitro* study of a human breast cancer cell line conducted by Monte *et al*. [11] revealed that the lectin protein isolated from okra could induce up to 63% cell growth inhibition. Accordingly, Wahyuningsih *et al*. [12] showed that the administration of an okra polysaccharide extract increased the immune response through the increase in TNF-α, IFN-γ, and Natural Killer (NK) cell activity in mice with inflammation caused by bacterial infection. Despite such evidence, a study of the activity of an ethanol extract of red okra pods as an anti-breast cancer agent has not been performed.

This study aimed to determine the potential of a red okra pod ethanol extract as an anti-breast-cancer agent in mice induced by *N*-methyl-*N*-nitroso urea (NMU). The anti-cancer activity was assessed in histological preparations of breast organs, CD4+/CD8+ T cells from spleen organs, and several essential cytokines involved in breast cancer, such as IL-1, IL-6, TNF-α, IFN-γ, TGF-β, and IL-10, in the blood serum of mice.

## Materials and Methods

### Ethical approval

The study was approved by Animal Care and Use Committee (ACUC) of Veterinary Faculty, Universitas Airlangga, Surabaya, Indonesia with approval number: 2.KE.057.04.2019.

### Study period and location

This study was conducted from June 2020 to September 2020. The experimental study was conducted in Animal Laboratory and Molecular Genetics Laboratory, Department of Biology, Faculty of Science and Technology, Universitas Airlangga.

### Materials and chemicals

Red okra pods were obtained from a traditional market in Surabaya, Indonesia. Red okra was identified by Balai Konservasi Tumbuhan, Kebun Raya Purwodadi, Indonesian Institute of Science (LIPI), Pasuruan, Indonesia with specimen vouchers (1205/IPH.06/HM/XI/2019) were deposited in the herbarium of Kebun Raya Purwodadi, LIPI, Pasuruan, Indonesia. Ethanol pro-analyze was purchased from Merck (Merck Millipore, Darmstadt, Germany). NMU, lyophilized powder, was purchased from Merck (Merck Millipore). Interleukin-6 (IL-6), IL-10, IL-1β, IL-17, TGF-β, and TNF-α Enzyme-linked Immunosorbent Assay (ELISA) kit was purchased from Biolegend (Biolegend, Inc., San Diego, USA). All other chemicals and solvents used in the experiments were analytical reagent grade.

### Preparation of the ethanolic extract of red okra pods (EEROP)

Fresh red okra pods were cleaned with distilled water, cut into small pieces, and air-dried for 7-14 days, according to Chen *et al*. [13], with modification. During the air-drying process, the red okra pods were not exposed to sunlight and the oven’s high temperature. First, dried pods were pulverized using an electric grinder, to obtain a fine powder. Next, 500 g of dried okra pod powder was macerated with 500 mL of ethanol overnight, at room temperature (25°C) with constant stirring. Subsequently, the extract was filtered and macerated again twice with 500 mL of ethanol. Next, to obtain EEROP in fine form, the extract was subjected to a rotary evaporator and freeze-dried.

### Animals

Female rats (*Rattus norvegicus*) (7-8 weeks old, 120-130 g) were purchased from the Faculty of Pharmacy, Universitas Airlangga, Surabaya, Indonesia. The rats were housed at ~20°C with a 12-h light/12-h dark cycle and were allowed free access to food and water during the experiments (*ad libitum*).

### Experimental design

Before the experiment, all rats were acclimatized for one week according to the animal care procedure of the ACUC of Veterinary Faculty, Airlangga University, Surabaya, Indonesia. These mice were randomly divided into six groups (n=5/group): (1) The KN group, as a normal control without any treatment; (2) the K−group, as a negative control exposed to MNU without EEROP administration; (3) the K+ group, as a positive control exposed to MNU and Methotrexate (an anti-cancer medicine); (4) the P1 group, with EEROP administration at 50 mg/kg body weight (BW) of; (5) the P2 group, with EEROP administration at 100 mg/kg BW; and (6) the P3 group, with EEROP administration at 200 mg/kg BW. A single dose of MNU (50 mg/kg BW) was injected intraperitoneally. Rats were incubated for 28 days, to ensure the development of breast cancer. Subsequently, EEROP was administered through oral gavage for 28 days. The rats were weighed 3 h after the last EEROP administration and sacrificed. Cardiac blood, spleens, and breasts were collected from the rats.

### Measurement of TGF-β and serum cytokine levels

Whole blood (3-4 mL) was collected from cardiac veins and centrifuged at 1006× *g* at 4°C for 10 min (Eppendorf, Germany); the supernatant contained the serum. The levels of TGF-β, IL-6, IL-10, IL-1β, IL-17, and TNF-α in the serum were analyzed using commercial ELISA kits (Bio Legend, Massachusetts, USA), according to the manufacturer’s protocol. Absorbance was measured using an ELISA reader (Thermo Fisher Scientific, USA) at 450 nm.

### CD4^+^ and CD8^+^ flow cytometric assay

Penicillin–streptomycin, collagenase type IV, and DNAse were added to mammary glands for 30 min. Subsequently, the mammary glands were washed in phosphate-buffered saline (pH 7.4) and centrifuged (Eppendorf, Germany) at 1789× *g* at 24°C for 8 min. This process aimed to obtain a single-cell suspension of the mammary gland. Then, the single-cell suspensions of the mammary gland were stained with anti-CD4-PerCP-Cy5.5 and anti-CD8-phycoerythrin at 4°C for 20 min to detect CD4+ and CD8+, respectively. Finally, CD4+ and CD8+ cells were measured using a flow cytometer (BD FACS Calibur, BD Bioscience, USA) and analyzed using FlowKo 7.6.

### Histology of the mammary gland

The histological examination of the mammary gland was conducted as per the method described by Kiernan [14]. First, the mammary gland was fixed with 10% formalin buffer phosphate, followed by washing and processing with a graded alcohol series (70%, 80%, and 96% absolute alcohol) and xylol I-II. Then, the organ was embedded in paraffin. Organ sectioning was performed using a microtome at a thickness of 4 μm. The organ tissues were stained with hematoxylin and eosin), and the histology of the mammary gland was observed under a light microscope. We observed connective tissue, lobular tissue, and myoepithelial cells on the lobules of mammary glands. According to mammary gland tumor pathology, we also determined the type of tumor that developed in the rats [15].

### Statistical analysis

Statistical analysis was performed by one-way analysis of variance followed by the Duncan *post hoc* test. All analyses were performed using the IBM Statistical Package for the Social Sciences Statistics software version 24.0 (IBM Corp., NY, USA). The results are presented as the mean±standard deviation. Significance was set at p<0.05.

## Results

### Level of transforming growth factor-β

**Figure-1:**
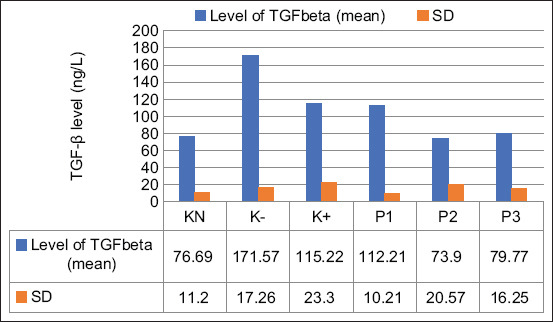
Effect of the ethanolic extract of red okra pods on the level of tumor growth factor-β (ng/L). KN=Normal control, K−=Negative control, K+=Positive control. P1, P2, and P3=Administrated with 50, 100, and 200 mg/kg body weight ethanolic extract of red okra pods. Each bar represents means±standard deviation (n=5). *p<0.05 compared to the negative control. **p<0.05 compared to negative and positive control groups.

The serum levels of TGF-β were significantly decreased in the P2 (73.9±20.57 ng/L) and P3 (79.77±16.25 ng/L) groups compared with negative control and positive control groups (p=0.05). The P2 and P3 groups showed no difference compared with the normal control groups (Figure-1).

### Level of serum cytokines

The serum levels of IL-6, IL-1β, and TNF-α were significantly decreased in the P1, P2, and P3 groups compared with the negative control groups (p=0.05) (Table-1). The P1 group exhibited the lowest level of IL-6 compared with the remaining groups (0.61±0.04 ng/L). Next, the P3 group exhibited the lowest level of IL-1β and TNF-α compared with the normal control and negative control groups.

**Table 1:** Effect of ethanolic extract of red okra pods on the level of cytokines.

Group	Cytokine				

	IL-6 (ng/L)	IL-10 (ng/L)	IL-17 (pg/mL)	IL-1β	TNF-α
KN	0.68±0.07	86.10±3.44	53.44±12.01	2187.86±172.44	47.67±9.31
K−	0.92±0.05*	115.04±9.13****	162.40±15.54	2695.36±257.10*	214.24±89.53****
K+	0.83±0.02*	98.04±1.94*	32.25±5.94****	2166.15±48.91	32.08±5.88**
P1	0.61±0.04***	90.43±5.13	36.68±6.77****	2660.53±123.73*	57.57±8.52***
P2	0.66±0.03**	92.76±3.92	30.62±9.00****	2070.61±235.91	41.36±10.82***
P3	0.70±0.05***	97.30±4.08*	32.29±8.84****	2021.90±133.07	37.28±8.25***

KN=Normal control, K−=Negative control, K+=Positive control, P1, P2, and P3=Administrated with 50, 100, and 200 mg/kg BW EEROP. Each bar represents means±SD (n=5).

* p<0.05 compared to normal control and negative control.

** p<0.05 compared to negative control and positive control.

*** p<0.05 compared to normal, positive and control groups.

**** p<0.05 compared to all groups, EEROP=Ethanolic extract of red okra pods, SD=Standard deviation, IL=Interleukin, TNF=Tumor necrosis factor

The serum levels of IL-10 in the P1, P2, and P3 groups were reduced compared with the normal control and negative control groups (p=0.05). The serum levels of IL-10 in the P1, P2, and P3 groups were lower than that detected in the positive control groups. The serum levels of IL-17 in the P1, P2, and P3 groups were significantly decreased compared with the normal control and negative control groups. Next, the serum levels of IL-17 in the P1, P2, and P3 groups were lower than those detected in the positive control groups (p=0.05). The IL-17 levels in the P1, P2, and P3 groups were comparable to the level of IL-17 in the positive control group.

### Activity of CD4+ and CD8+ T cells

**Figure-2:**
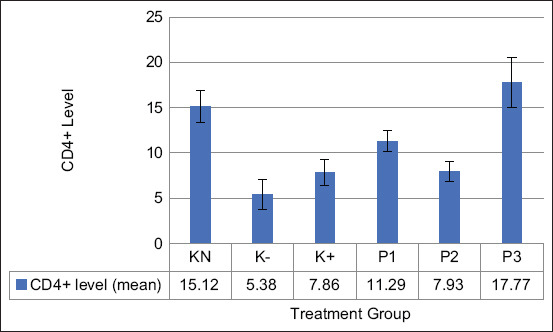
Effect of the ethanolic extract of red okra pods on the activity of CD4+ T cell. KN=Normal control, K−=Negative control, K+=Positive control. P1, P2, and P3=Administrated with 50, 100, and 200 mg/kg body weight ethanolic extract of red okra pods. Each bar represents means±standard deviation (n=5). *p<0.05 compared to the negative control. **p<0.05 compared to negative control and positive control. ***p<0.05 compared to all groups.

CD4+ T cells are immunocompetent cells that play a role in the specific immune response, whereas CD4+ T cells demonstrate a significant function in recognizing specific antigens. According to Figure-2, the activity of CD4+ T cells was significantly increased in the P1, P2, and P3 groups compared with the normal control and negative control groups (p=0.05). In addition, the P3 group showed the highest activity of CD4+ T cells compared with the remaining groups (17.77±2.73).

**Figure-3:**
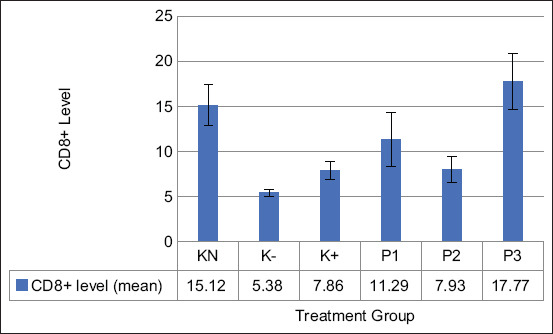
Effect of ethanolic extract of red okra pods on the level of CD8+ T cell activity. KN=Normal control, K−=Negative control, K+=Positive control. P1, P2, and P3=Administrated with 50, 100, and 200 mg/kg body weight ethanolic extract of red okra pods. Each bar represents means±standard deviation (n=5). *p<0.05 compared to the negative control. **p<0.05 compared to all groups.

The EEROP also affected CD8+ T-cell activity. According to Figure-3, the activity of CD8+ T cells was significantly increased in the P1 and P3 groups compared with the negative control groups (p=0.05). Furthermore, the P3 group showed the highest activity of CD8+ T cells compared with the remaining treatment groups (17.77±3.09).

### Histology of the mammary glands

**Figure-4:**
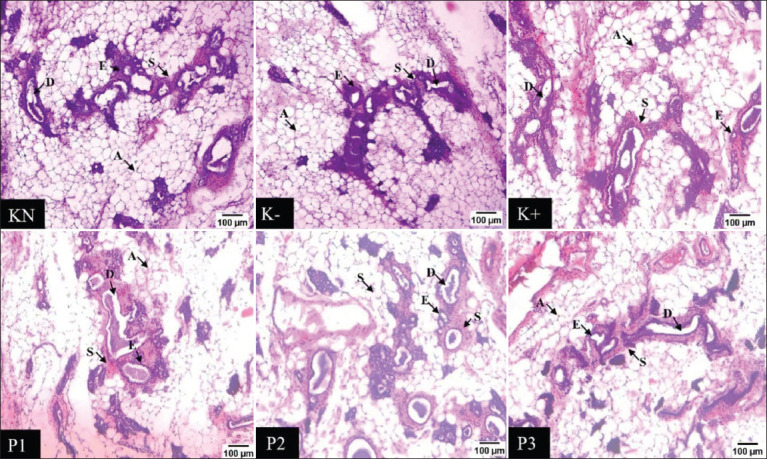
Effect of the ethanolic extract of red okra pods on the mammary gland histology. 100× magnification microscope. The arrows show the differences in mammary gland epithelial tissue. A=fat tissue, D=ducts, E=Epithelial tissue, S=Stroma. KN=Normal control; K−=Negative control, K+=Positive control. P1, P2, and P3=Administrated with 50, 100, and 200 mg/kg body weight ethanolic extract of red okra pods.

**Figure-5:**
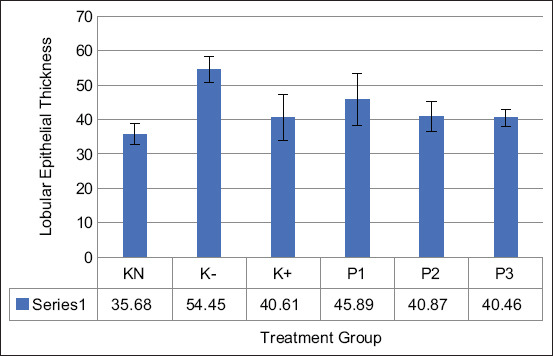
Effect of the ethanolic extract of red okra pods on the lobular epithelial thickness. KN=Normal control, K−=Negative control, K+=Positive control. P1, P2, and P3=Administrated with 50, 100, and 200 mg/kg body weight body weight ethanolic extract of red okra pods. Each bar represents means±standard deviation (n=5). *p<0.05 compared to the negative control.

Histological observations were carried out by measuring the thickness of the epithelium of the mammary glandular lobule. Based on the results of this study (Figures-4 and 5), cell proliferation occurred in the tissue section of epithelium in the MNU-induced group. Meanwhile, groups with the administration of EEROP showed significantly thinner epithelium compared to the normal control group (p=0.05).

## Discussion

This study aimed to investigate the potential of a red okra pod ethanolic extract as an anti-breast cancer agent in mice induced by NMU. Although the mechanism of breast cancer initiation is unknown, exposure to carcinogenic substances, such as MNU, can promote cancer development. Nitroso compounds act as carcinogenic agents by inducing tumor development in laboratory animals and are associated with human cancer. Humans can be exposed to MNU continuously through food (canned meat, cigarette smoke, beer, sauces, etc.), cosmetic products, domestic tools, and industrial–agricultural waste. MNU can produce the O^6^-alkilguanin compound, which exhibits mutagenic and carcinogenic characteristics. MNU-induced mammary gland carcinogenesis is a model that has been trusted to be almost identical to breast cancer in humans. This model is based on the origin of the tumor growth, exhibits local invasion, is associated with steroid hormones, and demonstrates the ability to change the expression of various genes, such as *Ras/Raf/mitogen-activated protein kinase (MAPK)*, *P13K/Akt*, *P53*, *e-kit*, *mTOR*, and *c-myc*.

MNU induces tumor development through initiation and promotion stages. Inflammation is one of the transition pathways in cancer development. Moreover, inflammation can occur after MNU exposure, as this agent can increase reactive oxygen species (ROS). In turn, ROS cause cellular oxidative stress, which leads to DNA damage and the formation of neoantigens, which increase the activity of cytokines and the immune system. Next, cytokines such as IL-10, TNF-α, and IL-6 can induce the nuclear factor kappa B (NF-κB) cascade reaction, which regulates inflammation, causes apoptosis inhibition, and decreases *p53* gene expression. A decrease in cell apoptosis and inhibited regulation of cell growth can cause uncontrolled proliferation. Therefore, cytokines and immune components become important factors in the promotion or inhibition of cancer development. One of the preventative measures for chronic inflammatory reactions is the modulation of the regulatory process through a substance that can act as an immunomodulator.

The TGF-β is a cytokine that is produced by cancer and stromal cells, including immune cells and fibroblasts. The present results demonstrated that EEROP administration at 100 and 200 mg/kg BW lowered the expression of TGF-β in a manner similar to that observed in the normal control group. Furthermore, a study conducted by Ouanouki *et al*. [16] showed that anthocyanin derivate, namely, anthocyanidins, can reduce TGF-β expression in glioblastoma cancer cells. The inhibition of TGF-β signaling may prevent cancer metastasis by inhibiting the epithelial-mesenchymal transition (EMT). In addition, researchers reported that anthocyanins from black rice suppressed the RAS/RAF non-Smad MAPK entry line to produce inhibition of MDA-MB 453, which can prevent metastasis.

Cytokines are small proteins that are produced by cells such as T-helper cells, NK cells, and macrophages and regulate the immune response and inflammation [17]. The present results showed that the EEROP is potentially an anti-inflammatory agent by inhibiting pro-inflammatory cytokines such as NF-κB, TNF-α, and IL-6. Suppression of the inflammatory molecule occurs by suppressing the transcription of NF-κB [18]. Furthermore, a study conducted by Zhang *et al*. [19] reported similar results. A significant decrease was found in the serum levels of IL-6, IL-1β, and TNF-α after the administration of an apigenin extract as an anti-inflammatory material against lipopolysaccharide induction. Furthermore, Verma *et al*. [20] stated that IL-6, IL-1β, and TNF-α are significantly decreased after the administration of *Aegle marmelos* against MNU exposure.

EEROP contain flavonoids such as quercetin and rutin, flavanones such as apigenin and luteolin, and anthocyanin as a pigment material. IL-6 is a pro-inflammatory cytokine that is produced when inflammation, infection, and tumors occur [21]. In turn, IL-1β serves as a master of cytokines, which could further induce the expression of other pro-inflammatory cytokines, such as IL-6 and TNF-α, chemokines, adhesion molecules, and other molecules associated with inflammation, to strengthen the inflammatory response [22]. The primary regulation of pro-inflammatory gene expression is the activation of NF-κB [23]. NF-κB is vital for the regulation of inflammatory reactions in inflammation or tumors. Inhibition of NF-κB activation is an important mechanism used by flavonoids to inhibit inflammation. Inhibition of NF-κB activation will decrease the production of cytokines, such as TNF-a, IL-6, and IL-1ß [24].

Next, other cytokines include IL-10 and IL-17. The present results showed a decrease in the levels of IL-10 and IL-17. This result was in line with the study conducted by Alessandra-Perini *et al*. [25], who stated that an ethanolic extract of *Euterpe oleracea* could decrease IL-10 serum levels significantly. Furthermore, the level of IL-10 is correlated with the inflammation process. The development and sustainability of breast tumors are mediated by an imbalance between pro-inflammatory and anti-inflammatory cytokines [26]. In turn, a decrease in IL-10 serum levels will help inhibit breast tumor growth. IL-10 and IL-6 can activate the phosphorylation of STAT-3 at the Tyr705 amino acid position through the activation of JAK [27]. The EEROP contained anthocyanin and quercetin.

Quercetin can dephosphorylate STAT3 at Tyr705 and Ser727. STAT3 can positively affect the IL-6 and IL-10 concentrations through positive feedback [28]. This implies that quercetin affects the concentration of IL-6 and IL-10 and helps modulate the levels of these cytokines. Regarding IL-17 levels, Meng *et al*. [29] also stated that a quercetin extract decreased IL-17 levels in rats with chronic prostate inflammation. Quercetin can suppress the activity of NF-κB and block the MAPK pathway. The downregulation of pro-inflammatory cytokines, such as IL-1β, IL-6, and TNF-α, can suppress the differentiation of CD4+ T cells into T-helper 17 cells (Th17), which are a source of the IL-17 cytokine.

Based on the data on the levels of cytokines in the serum, the EEROP at various doses could be used as an immunomodulator by suppressing the levels of TGF-β, IL-6, IL-10, IL-1β, IL-17, and TNF-α significantly. The bioactive compounds of EEROP, especially flavonoids, demonstrate the potential to act as immune regulators in the MNU-induced tumor microenvironment in mice. However, the crude extract containing flavonoids will give slightly different results than the pure flavonoid extract because of the interaction between the flavonoids and other compounds of the crude extract.

CD4+ T cells and CD8+ T cells are immunocompetent cells that play a role in the specific immune response. The present results demonstrated the potential of EEROP to increase the amount of CD4+ and CD8+ T cells. The same result was reported in the study of Ajaghaku *et al*. [30]. Furthermore, this study showed the positive effect of quercetin-3-*O*-rutinoside to increase the activity of CD4+ T cells in mice with toxoid tetanus. Quercetin-3-*O*-rutinoside is a type of quercetin that plays a prominent immunological role in medicinal plants [31]. Quercetin can stimulate the proliferation of T-cell lymphocytes [30]. Long-term quercetin administration was reported to increase the proliferation of immune cells in the splenic organ and significantly impact the CD4+/CD8+ response to suppress the induction of the immune system by radiation [32]. The upregulatory effect on the CD4+ population of adaptive immunity by quercetin may positively enhance the function of other cells of adaptive immunity, as well as other immune cells.

Furthermore, a previous study conducted by Novitskiy *et al*. [33] reported that apigenin treatment increased the activity of CD8+ T cells and decreased the percentage of regulatory T cells. Cancer cells may survive by maintaining the activity of regulatory T cells and inhibiting effector T-cell activity. Regulatory T cells could suppress the anti-tumor immune response. The EEROP contain quercetin and apigenin, which increase the Ikaros transcription factor. This transcription factor is vital for the development of lymphocytes, especially T cells. Mishra *et al*. [34] stated that the downregulation of Ikaros was correlated with a significant CD4+ and CD8+ activity decrease. The increased activity of CD8+ T cells can afford an improved immune response to infection or diseases triggered by an immune response.

The present study examined the histology of the mammary gland. Treatment with EEROP yielded a thinner epithelium, and thickening of epithelium tissue was observed in the MNU-induced group without EEROP administration. The thickening of the epithelium tissue was caused by cell proliferation. Cell proliferation begins from chronic inflammatory regulation in the NF-κB pathway, which increases cyclin D1 in the cell cycle. In turn, increased cyclin D1 may lead to progression from the G1 phase to the S phase. In addition, the TGF-β cytokine can trigger the EMT, leading to cell proliferation. EMT was increased along with matrix metalloproteinase (MMP)-2 and MMP-9. Furthermore, EMT activates epidermal growth factor receptors (EGFR), which activate the PI3K signaling pathway. Vascular endothelial growth factor (VEGFR)-2 plays an essential role in the activation of the PI3K and p38 MAPK signaling pathways, which are activated by cancer cells, including tumor mammary gland growth. According to the study conducted by Chien *et al*. [35], quercetin can inhibit tumor growth in the mammary glands of DMBA-induced mice. The EEROP also contains quercetin. Quercetin can prevent cell proliferation by significantly downregulating vimentin, N-cadherin, MMP-2, MMP-9, p-EGFR, VEGFR-2, p-PI3K, Akt, and pGSK3ß. Because of this function, quercetin can slow down carcinogenesis.

## Conclusion

It can be concluded that the EEROP could modulate the immune response by (1) downregulating IL-1, IL-6, TNF-α, IFN-γ, TGF-β, and IL-10, to prevent the overexpression of pro-inflammatory cytokines, (2) increasing the activity of CD4+ and CD8+ T cells, to improve the immune response against cancer, and (3) protecting the epithelium of mammary gland from thickening because of cancer cell proliferation. Thus, this study suggests that the EEROP could act as an anti-cancer agent by modulating the immune response. However, this study may exhibit limitations. Additional studies using a nanoparticle system are necessary for the optimal function of bioactive compounds.

## Authors’ Contributions

SPAW, DW, MP, and RWW: Conceptualization and designed the study. SPAW, MP, and DWA: Experimentation and data collection. DW and FRPD: Data analysis. SPAW and MP: Drafted and revised the manuscript. All authors have read and approved the final manuscript.
